# Socioeconomic and Clinical Predictors of Mortality in Patients with Acute Dyspnea

**DOI:** 10.2147/OAEM.S277448

**Published:** 2021-03-25

**Authors:** Torgny Wessman, Rafid Tofik, Thoralph Ruge, Olle Melander

**Affiliations:** 1 Department of Emergency Medicine, Skåne University Hospital, Malmö, Sweden; 2 Department of Clinical Sciences, Lund University, Malmö, Sweden; 3 Department of Internal Medicine, Skåne University Hospital, Malmö, Sweden

**Keywords:** acute dyspnea, emergency department, risk factor, immigrant, smoking, socioeconomic status, mortality, comorbidity, METTS

## Abstract

**Background:**

Factors predicting long-term prognosis in patients with acute dyspnea may guide both acute management and follow-up. The aim of this study was to identify socioeconomic and clinical risk factors for all-cause mortality among acute dyspnea patients admitted to an Emergency Department.

**Methods:**

We included 798 patients with acute dyspnea admitted to the ED of Skåne University Hospital, Malmö, Sweden from 2013 to 2016. Exposures were living in the immigrant-dense urban part of Malmö (IDUD), country of birth, annual income, comorbidities, smoking habits, medical triage priority and severity of dyspnea. Mean follow-up time was 2.2 years. Exposures were related to risk of all-cause mortality using Cox proportional hazard model.

**Results:**

During follow-up 40% died. In models adjusted for age and gender, low annual income, previous or ongoing smoking, certain comorbidities, high medical triage priority and severe dyspnea were all significantly associated with increased mortality. After adjusting for age, gender and all significant exposures, the lowest quintile of income, ongoing or previous smoking, history of serious infection, anemia, hip fracture, high medical triage priority and severe dyspnea significantly and independently predicted mortality. In contrast, neither country of birth nor living in IDUD predicted a mortality risk.

**Conclusion:**

Apart from several clinical risk factors, low annual income predicts two-year mortality risk in patients with acute dyspnea. This is not the case for country of birth and living in IDUD. Our results underline the wide range of mortality risk factors in acute dyspnea patients. Knowledge of patients’ annual income as well as certain clinical features may aid risk stratification and determining the need of follow-up both in hospital and after discharge from an ED.

## Background

Shortness of breath is a common symptom in Emergency Department (ED) patients. At the ED at Skåne University Hospital (SUS) in Malmö, Sweden, in 2018 out of 75,535 patients´ admissions 4,562 (6%) patients had shortness of breath as the primary admission complaint (statistics from patient ledger, the hospital database of patient visits, Database Region Skåne, Malmö, Sweden, personal communication, 2018). This makes shortness of breath the third most common cause of ED visits, which is similar in other ED:s in Europe.^1^ Many different diseases and conditions cause acute shortness of breath, which we henceforth will refer to as acute dyspnea. Dyspnea is one of the most frequent complaints in the elderly^2^ making it a growing problem with an increasingly older population. The most common causes are cardiovascular diseases (CVD) and diseases of the respiratory system.^3^ The literature contains a large volume of disease and diagnosis-specific studies. However, patients arriving at an ED usually present a symptom rather than a diagnosis. It is therefore necessary for emergency physicians in an acute setting to make decisions regarding the intensity of treatment, level of care, as well as make a relevant plan for follow-up at hospital discharge primarily on the presentation of a symptom rather than a pre-set diagnosis. Symptoms of acute dyspnea are associated with diseases with high mortality.^3^,^4^ Identifying patients at high risk who need more intense treatment and follow-up during their relatively short time at the ED is challenging. The use of different medical triage systems in the ED setting is now widely used and evaluated for risk stratification.^5–7^ Knowledge about the patients’ previous medical history and comorbidities is important information. The use of combined scores predicting the risk of poor outcome has been useful in other settings.^8–13^ The identification of risk factors for poor outcome is important in decision-making. Many cardiovascular and respiratory diseases are attributable to lifestyle and socioeconomic risk factors and such factors may also be involved in compliance to treatment and the prognosis after an acute event. In a previous retrospective study of 184 dyspnea patients who visited the ED of (SUS) Malmö, Sweden in 2007, we found that patients from the first and second generation immigrant-dense part of the city of Malmö (IDUD) had a significantly higher five-year mortality compared to the other part of Malmö, ie, “Swedishborn-dense urban districts” (SDUD), regardless of several other risk factors.^14^ The reason for this association was unclear. However, the results pointed to a lower socioeconomic status (SES) in the IDUD part of Malmö as a possible cause. Based on our findings in the previous study, we aimed to identify clinical and socioeconomic factors, as well as living in the IDUD part of Malmö, as predictors of long-term mortality in a large and prospective study of unselected patients seeking care at the ED due to acute dyspnea. We hypothesized that patients from the IDUD half of Malmö as defined in 2007 (Necmi Incegül, city of Malmö, Department of Planning, personal communication, 2007) as well as country of birth would affect mortality. Our hypothesis was also that low annual income, smoking, presence of certain comorbidities, as well as high METTS priority and severe dyspnea would affect mortality.

## Methods

**Figure 1:**
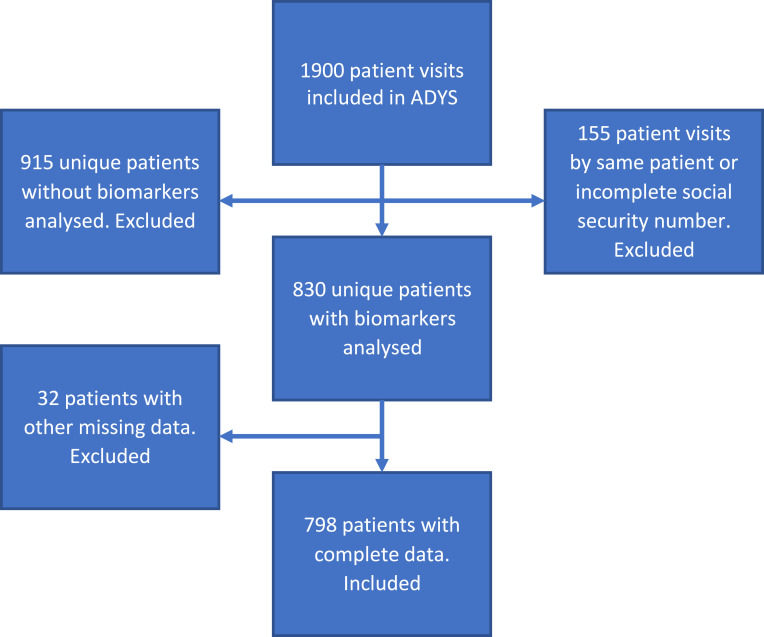
Flow-chart cohort from ADYS.

For our current study, we used a subgroup of 798 patients from the “Acute Dyspnea Study” (ADYS) (Figure 1). The inclusion criteria for ADYS were being patients with acute dyspnea admitted to the ED of SUS Malmö, Sweden, and age above 18 years. In total 1900 patient-visits were included between March 2013 and December 2018. Patients with multiple visits were only included once, and patients with unknown address or missing social security number were excluded, in total 255 patient admissions. A total of 115 patients were registered either at an unknown habitat in Malmö or were not registered as living in Malmö. Most of the patients who were not registered as living in Malmö were patients from communities just outside Malmö, from an area with a similar demography. As the group of patients who were not registered as living in Malmö was so large, they were included in the analyses, however, for the exposure of IDUD vs SDUS they were treated as a separate category. This means a remaining cohort of 1745 unique patients in ADYS (Figure 1). Patients visiting the ED with acute dyspnea as their primary complaint on arrival were informed about the study and asked for their written informed consent. This study complies with the Declaration of Helsinki. Patients 18 years or older were included daytime working days by a research nurse. Critically ill patients who were directly transferred to an intensive care unit (ICU) from the resuscitation room were excluded, as were patients with lower degrees of consciousness. A research nurse collected information from the patients´ medical hospital records, and patients were interviewed about their health, medication, symptoms, social situation, etc. according to a standardized and approved questionnaire (see supplement).

### Study Population and Plan

In the current study, we included the first 798 patients (included between 6 March 2013 and 20 January 2016). The patients included during this period were originally 830, but we lacked information on income, smoking habits, country of birth, METTS^15^ triage priority or level of dyspnea on 32 patients leaving us with a total cohort of 798 patients (Figure 1). In 2017, a data file containing data on these 798 patients was sent in a coded file to Statistiska Centralbyrån (SCB), Statistics Sweden (statistics concerning annual income from Statistiska Centralbyrån, Statistics Sweden, personal communication, 2017). At SCB, the file was decoded and SCB added information on an individual basis regarding income from 2012 to 2015. The file was then sent to the Swedish National Board of Health and Welfare (Socialstyrelsen), SoS, which used the code key from SCB to add information about cause of death until 31 December 2105 and date of death until 26 July 2017, ie, end of the follow-up period (Death register & Patient´s visits register, Socialstyrelsen, National Board of Health and Welfare, Sweden, personal communication, 2017). The Cause of Death Register at SoS is updated more slowly than the all-cause Death Date register itself. The Cause of Death Register lags approximately 2 years vs information on all-cause death, which explains the differences in number of patients with information of date of death vs information on cause of death. As our data file is anonymized, as required by Swedish law, it is not possible to afterwards supplement with later data from the Swedish Cause of Death Register.

### Follow-Up

The cohort was observed for mortality with a follow-up time until 26 July 2017, which means a mean follow-up time of 2.2 ±1.3 years. The end point of this study was all-cause mortality.

### Exposures

From the patient’s address, we registered the district of Malmö in which they lived. The IDUD half of Malmö comprises the five urban districts with the highest number of immigrants in 2007. The SDUD half of Malmö comprises the five urban districts with the highest number of Swedishborn inhabitants in 2007. A third group comprised patients living outside Malmö or who had missing information about where they lived. We used income during the year prior to visiting the ED as exposure, assuming it would be a better marker of overall SES, rather than the same year as the patient visited the ED, as the disease itself may have affected income during the same year and after. Country of birth was based on the questionnaire in which we asked every patient whether they were born in Sweden, outside of Sweden but within the EU, or outside the EU. Regarding smoking, patients were asked if they were present smokers, previous smokers or never smokers. The variable “ever smoking” was defined as either ongoing or previous smoking. The presence of comorbidities was also based on the patients’ answers in the questionnaire. We registered the medical priority according to METTS^15^ given to the patients on arrival by a research nurse. METTS priority 1 (red) are patients with such a severe condition that they must be immediately attended to by a team of emergency doctors and nurses, often in the resuscitation room. METTS priority 2 (orange) patients have potentially unstable vital parameters and must be examined by an emergency doctor and nurse within 15–30 minutes. METTS priority 3 (yellow) patients have stable vital parameters on arrival and can wait for up to one hour to see a doctor. METTS priority 4 (green) patients have the lowest priority in the system and sometimes have to wait for up to four hours to see a doctor. Finally, a nurse assessed the level of dyspnea for all patients, using a similar scale as the NYHA classification.^16^ Dyspnea level 1 patients had no dyspnea at all, whereas level 2 patients had slight dyspnea symptoms and level 3 patients experienced dyspnea during physical activity. Finally, level 4 patients also experienced dyspnea at rest.

### Statistics

Data are presented as median (interquartile range) or mean (± SD), depending on the presence or absence of normal distribution of data. Group-wise differences of continuous variables were compared using ANOVA or the Kruskal–Wallis test when appropriate. Categorical variables were compared between groups using the Chi-2 test. We used the Cox proportional hazards model to relate exposures to variables in relation to mortality in a first model adjusted for age and gender. In a second model we adjusted for all exposures that were significant in the first model in addition to age and gender. A two-tailed significance level of (p<0.05) was considered statistically significant.

## Results

Patient characteristics on arrival are summarized in Table 1. The mean age was 69 years (SD ± 18.4) with 66% of patients being above 65 years of age. 46% were male and 72% were born in Sweden. 55% arrived via ambulance, 43% had received a present or previous diagnosis of hypertension, 33% cardiac heart failure (CHF), 31% coronary heart disease (CHD), 29% chronic obstructive pulmonary disease (COPD) and 28% atrial fibrillation (AF). Clinical background features and vital parameters are shown in Table 2. Of the patients 7% had tachycardia, 18% had a respiratory rate >28/min and 19% were hypoxic with an oxygen saturation <90%. Almost all (94%) patients had an ECG taken, and 53% of the patients had a chest X-ray taken. More than half (57%) of the patients were admitted to a ward for in-hospital care. During the 2.2 years of follow-up until 2017–07-25, 334 (40%) of the patients died. We only have access to the specific cause of death in 45 patients for 30-days mortality and in 222 patients with follow-up until 2015–12-31. The three main causes of death during full follow-up were cardiovascular (40%, stroke included), neoplasms and malignancies (21%) and COPD and other pulmonary diseases (19%, pneumonia included). Similarly, the three main causes of death after 30-days were cardiovascular (40% stroke included), neoplasms and malignancies (24%) and COPD and other pulmonary diseases (10%, pneumonia included).

**Table 1:** Patient Baseline Characteristics (n=798)

Variables	Result
Age, years, mean (± SD)	70 (± 18)
Age >65 years, N (%)	542 (68%)
Gender (Male), N (%)	367 (46%)
**Region of birth**	
Sweden, N (%)	577 (72%)
EU (not Sweden), N (%)	155 (19%)
Outside EU, N (%)	66 (8%)
**Comorbidities (previous/ongoing), N (%)**	
Hypertension	340 (43%)
Cardiac heart failure	260 (33%)
Coronary artery disease	248 (31%)
Chronic obstructive pulmonary disease	231 (29%)
Atrial fibrillation	225 (28%)
Infection	219 (27%)
Obesity	173 (22%)
Anaemia	143 (18%)
Diabetes	143 (18%)
Cancer	140 (18%)
Pulmonary embolism	94(12%)
Asthma	86 (11%)
Stroke	81 (10%)
Renal disease	69 (9%)
Rheumatic disease	41 (5%)
Restrictive pulmonary disease	41 (5%)
Depression	37 (5%)
Anxiety	31 (4%)
Hip fracture	26 (3%)
Dementia	24 (3%)
Other pulmonary disease	15 (2%)
Neuromuscular disease	4 (0,5%)
**Smoking**	
Ongoing or previous smoker, N (%)	563 (71%)
Never smoker	235 (29%)
**Arrival mode**	
Ambulance, N (%)	437 (55%)
Alarm	74 (9%)
**Inhabitant of Malmö, N (%)**	716 (90%)

**Table 2:** Clinical Baseline Features and Vital Parameters (n=798)

Variables	Result
**Pulse rate**	
Bpm mean (range)	91 (22–225)
Median bpm (IQR)	88 (76–103)
Rate >120 bpm, N (%)	58 (7.3%)
**Respiratory rate**	
Breaths/min, mean (range)	24 (9–60)
Breaths/min, median (IQR)	23 (20–28)
Rate >28/min, N (%)	142 (17.8%)
**Systolic blood pressure**	
mmHg, mean (range)	145 (12–250)
mmHg, median (IQR)	140 (127–160)
<100 mmHg, N (%)	15 (1.9%)
**Diastolic blood pressure**	
mmHg, mean (range)	81(28–156)
mmHg, median (IQR)	80 (70–90)
**Pulse Oximetry**	
%, mean	93 (52–100)
%, median (IQR)	(95 91–98)
<90**%**, N (%)	148 (18.5%)
**Temperature**	
°C, mean (range)	36.9 (30.5–39.1)
°C, median (IQR)	36.9 (36.5–37.3)
Temp <35 or >38 °C, N (%)	13 (1.6)
**BMI** kg/m^2^, median (IQR)	25.5 (22.6–30.0)
**Need of interpreter**, N (%)	42 (5.3%)
**ECG**, N (%)	750 (94%)
**X-ray** (pulmonary), N (%)	421 (53%)
**ECHO**, N (%)	16 (2%)
**CT scan** thorax, N (%)	101 (13%)
**Preliminary diagnose arrival**, N (%)	
Dyspnea	792 (99,2%)
Heart failure	0
Acute coronary syndrome	1 (0.1%)
COPD	0
Pneumonia	0
Thromboembolic disease	0
Anxiety	0
Other disease	4 (0.5%)
System missing	1 (0.01%)
Total	798 (100%)
**Admitted** to hospital ward, N (%)	452 (57%)

**Abbreviations:** Bpm, beats per minute; IQR, interquartile range; BMI, body mass index; ECG, electrocardiogram; ECHO, echocardiogram; CT scan, computer scan.

### Socioeconomic Factors

Median annual income for our patients in the year prior to inclusion was 177 480 SEK (IQR: 125 682–228 435 SEK), 10 SEK = 1 euro. For comparison, the median annual income for inhabitants in Malmö >18 years of age for 2013 was 197 600 (IQR, 72,900–321 200) SEK) (Necmi Incegul, Statistics Malmö, personal communication, 2020). We divided the cohort of 798 individuals into quintiles of annual income (highest income for quintile 1). The median income (interquartile range) was SEK 340,000 (SEK 289,000–427 000) in quintile 1; SEK 217,000 (SEK 205,000–230,000) in quintile 2; SEK 177,000 (SEK 169,000–187 000) in quintile 3; SEK 138,000 (SEK 125,000–148,000) in quintile 4; SEK 83,000 (SEK 9,000–102,000) in quintile 5. Table 3 shows the baseline clinical characteristics of patients in income quintiles 1–5. Individuals with the lowest and highest income had the lowest age in comparison with the three central quintiles. Moreover, the percentage of males increased with income. Since age and gender are established factors that influence the mortality rate, we first adjusted all mortality analyses for age and gender. After adjusting for gender and age, there was a significantly increased mortality of 63% for the middle income quintile, 61% for the second-lowest income quintile and a 74% increased mortality rate in the lowest income quintile in comparison with the highest (reference) income quintile (Table 4). The relationship between income and mortality risk was linear (p-value for linear trend over quintiles = 0.023). There were no statistical differences in mortality risk between either the IDUD vs SDUD parts of Malmö or between countries of birth (Table 4).

**Table 3:** Baseline Characteristics in Relation to Income Quintiles (n=798)

	Income Quintile 1 n=157	Income Quintile 2 n=160	Income Quintile 3 n=161	Income Quintile 4 n=161	Income Quintile 5 n=159
Income (SEK) in the year prior to inclusion, mean (range)	402519 (253284–1828004)	218856 (197052–253102)	178473 (160560–196764)	137516 (111408–160524)	61062 (0–110915)
Age (years) mean (± SD)	60 (± 19)	73 (± 16)	75 (± 14)	77 (± 14)	63 (± 21)
Male gender (N (%))	90 (57)	89 (56)	89 (55)	36 (22)	63 (40)
SDUD (N (%))	68 (43)	65 (41)	70 (43)	76 (47)	70 (44)
IDUD (N (%))	67 (43)	71 (44)	75 (47)	68 (42)	62 (39)
Other UD (N (%))	22 (14)	24 (15)	16 (10)	17 (11)	27 (17)
Country of birth (born in Sweden (N (%))	131 (83)	129 (81)	120 (74)	127 (79)	70 (44)
Country of birth (born in Europe but not in Sweden (N (%))	17 (11)	24 (15)	38 (24)	28 (17)	48 (30)
Country of birth (born outside Europe (N (%))	9 (6)	7 (4)	3 (2)	6 (4)	41 (26)
Smoking ongoing and previous (N (%))	104 (66)	113 (71)	135 (84)	103 (64)	108 (68)
METTS priority 1 (N (%))	7 (4)	23 (14)	20 (12)	27 (17)	17 (11)
METTS priority 2 (N (%))	43 (27)	58 (36)	64 (40)	56 (35)	39 (24)
METTS priority 3 (N (%))	91 (58)	67 (42)	71 (44)	69 (43)	87 (55)
METTS priority 4 (N (%))	16 (10)	12 (8)	6 (4)	9 (6)	16 (10)
Dyspnea level 1 (N (%))	64 (41)	37 (23)	34 (21)	34 (21)	50 (31)
Dyspnea level 2 (N (%))	50 (32)	58 (36)	63 (39)	58 (36)	61 (38)
Dyspnea level 3 (N (%))	19 (12)	28 (18)	27 (17)	30 (19)	17 (11)
Dyspnea level 4 (N (%))	24 (15)	37 (23)	37 (23)	39 (24)	31 (20)

**Abbreviations:** SDUD, Swedishborn-dense urban districts; IDUD, immigrant-dense urban districts; Other UD, other urban district (outside of Malmö or missing); METTS priority 1, highest priority, immediately to resuscitation room; METTS priority 2, unstable or potentially unstable vital parameters; METTS priority 3, currently stable vital parameters; METTS priority 4, lowest priority, stable patient; Dyspnea level 1, no symptoms; Dyspnea level 2, dyspnea during light exercise; Dyspnea level 3, dyspnea during heavy exercise; Dyspnea level 4, dyspnea at rest.

**Table 4:** Model 1: Single Models Cox Proportional Hazards Regression, Age and Gender Adjusted, for Mortality at Full Follow-Up (n=798)

IDUD	HR	95% CI	P-value
SDUD	1.0	Ref.	
IDUD	0,829	0,657–1,048	n.s.
Other UD (outside Malmö+missing)	1,073	0,740–1,558	n.s.
**Country of birth**			
Born in Sweden	1.0	Ref.	
Born in EU but not Sweden	1,041	0,785–1,379	n.s.
Born outside of the EU	0,668	0,352–1,265	n.s.
**Yearly income quintiles (Q)**			
Highest income Q	1.0	Ref.	
Second highest income Q	1,476	0,974–2,236	n.s.
Middle income Q	1,629	1,082–2,452	0,019
Second lowest income Q	1,609	1,052–2,460	0,028
Lowest income Q	1,739	1,119–2,703	0,014
**Smoking**			
Ever smoke (present or previous)	1,570	1,207–2,042	0,001
**Comorbidities**			
Pulmonary embolism	0,923	0,667–1,278	n.s.
Infection	1,667	1,329–2,090	<0,0001
Anaemia	1,673	1,310–2,138	<0,0001
Cancer	1,163	0,897–1,507	n.s.
Obesity	0,812	0,612–1,077	n.s.
Diabetes	1,347	1,036–1,751	0,026
Hypertension	0,945	0,756–1,181	n.s.
Stroke	1,118	0,817–1,531	n.s.
Dementia	1,064	0,632–1,790	n.s.
Anxiety	0,969	0,531–1,771	n.s.
Depression	1,434	0,876–2,346	n.s.
Renal disease	1,689	1,242–2,299	0,001
Hip fracture	1,775	1,116–2,823	0,015
Coronary artery disease	1,282	1,021–1,609	0,033
Congestive heart failure	1,548	1,233–1,944	0,0002
Atrial fibrillation	1,159	0,922–1,456	n.s.
COPD	1,474	1,178–1,846	0,001
Asthma	1,006	0,694–1,459	n.s.
Restrictive pulmonary disease	1,590	1,047–2,416	0,030
Other pulmonary disease	2,169	1,148–4,099	0,017
Neuromuscular disorder	1,163	0,288–4,699	n.s.
Rheumatic disorder	0,871	0,547–1,386	n.s.
**METTS priority on arrival**			
METTS priority 4	1.0	Ref.	
METTS priority 3	1,577	0,768–3,239	n.s.
METTS priority 2	3,079	1,506–6,298	0,002
METTS priority 1	3,645	1,732–7,674	0,001
**Dyspnea level on arrival**			
Unaffected	1.0	Ref.	
Slight dyspnea	1,553	1,069–2,255	0,021
Heavy dyspnea	2,265	1,514–3,390	<0,0001
Dyspnea at rest	3,170	2,177–4,616	<0,0001

**Abbreviations:** HR, hazard ratio; CI, confidence interval; SDUD, Swedishborn-dense urban districts; IDUD, immigrant-dense urban districts; Other UD, other urban district (outside of Malmö or missing); METTS priority 1, highest priority, immediately to resuscitation room; METTS priority 2, unstable or potentially unstable vital parameters; METTS priority 3, currently stable vital parameters; METTS priority 4, lowest priority, stable patient; Dyspnea level 1, no symptoms; Dyspnea level 2, dyspnea during light exercise; Dyspnea level 3, dyspnea during heavy exercise; Dyspnea level 4, dyspnea at rest.

### Clinical Factors

As shown in Table 4, during the full follow-up time ever smokers compared to never smokers had a significant increased mortality risk as well as patients with increasing METTS scores (priority 1 and priority 2). There appeared to be a linear association between the level of dyspnea and mortality risk. Patients with ongoing or previous diseases, such as pneumonia or other serious infection, anemia, diabetes, renal disease, hip fracture, coronary artery disease, cardiac heart failure, COPD, restrictive pulmonary disease and other pulmonary diseases, had significantly increased mortality risk in gender and age-adjusted models.

### Multivariate Analyses

When all the exposures that were significantly related to mortality in age and gender-adjusted models, ie, annual income, smoking, comorbidities, METTS priority and dyspnea level on arrival were entered simultaneously into a multivariate model in addition to age and gender, there was still a significant and independent increase in mortality risk in patients with the lowest vs the highest income quintile, previous and ongoing vs never smokers, METTS priority 2 and dyspnea level 4, previous or ongoing pulmonary or other serious infection, anemia, hip fracture, (Table 5). Additionally, we performed a multivariate model adjusted for all covariates, regardless of whether they displayed significant relationship with mortality or not in age and sex adjusted analyses, which gave similar results (data not shown).

**Table 5:** Multivariate Cox Proportional Hazards Regression for Mortality at Full Follow-Up, with Adjustments for Age and Gender and All Variables Significant in Table 4 (n=798)

	HR	95% CI	P-value
Age	1,052	1,041–1,064	<0,0001
Gender	0,799	0,620–1,030	n.s.
**Yearly income quintiles (Q)**			
Highest income Q	1,0	Ref.	
Second highest income Q	1,297	0,845–1,990	n.s.
Middle income Q	1,220	0,790–1,885	n.s.
Second lowest income Q	1,374	0,877–2,152	n.s.
Lowest income Q	1,644	1,038–2,603	0,034
**Comorbidities**			
Congestive heart failure	1,243	0,969–1,595	n.s.
COPD	0,986	0,771–1,261	n.s.
Restrictive pulmonary disease	1,465	0,951–2,256	n.s.
Infection	1,400	1,106–1,773	0,005
Other pulmonary disease	1,745	0,884–3,446	n.s.
Anaemia	1,529	1,185–1,971	0,001
Diabetes	1,205	0,914–1,589	n.s.
Renal failure	1,363	0,976–1,903	n.s.
Coronary artery disease	0,952	0,736–1,230	n.s.
Hip fracture	1,827	1,131–2,952	0,014
**Smoking**			
Previous and ongoing	1,432	1,087–1,885	0,011
**Dyspnea level**			
Dyspnea level 1	1,0	Ref.	
Dyspnea level 2	1,053	0,712–1,556	n.s
Dyspnea level 3	1,464	0,958–2,237	n.s
Dyspnea level 4	1,774	1,154–2,727	0,009
**METTS priority**			
METTS priority 4	1.0	Ref.	
METTS priority 3	1,334	0,64–2,756	n.s
METTS priority 2	2,200	1,057–4,580	0,035
METTS priority 1	2,092	0,951–4,601	n.s

**Abbreviations:** HR, hazard ratio; CI, confidence interval; METTS priority 1, highest priority, immediately to resuscitation room; METTS priority 2, unstable or potentially unstable vital parameters; METTS priority 3, currently stable vital parameters; METTS priority 4, lowest priority, stable patient; Dyspnea level 1, no symptoms; Dyspnea level 2, dyspnea during light exercise; Dyspnea level 3, dyspnea during heavy exercise; Dyspnea level 4, dyspnea at rest.

## Discussion

The key findings in this study were that SES in the form of low annual income the year prior to admission, as well as clinical factors in the form of ongoing or previous smoking, multiple comorbidities, high METTS priority and increased dyspnea level on arrival all are independent risk factors for increased mortality among acute dyspnea patients. Many population-based studies have examined and showed relationships between low socioeconomic status (SES) and increased mortality.^17^ In our previous study^14^ we found that a low annual income, high METTS priority and an increasing dyspnea level on arrival predicts mortality. To the best of our knowledge, our previous study^14^ and the present study are the first studies to show a link between low annual income and mortality in patients with acute dyspnea. A link between income and health status is supported by a large amount of evidence in a non-acute setting, ie, in studies of population health.^18^,^19^ There is evidence of a strong relationship between a society’s income distribution and the life expectancy of its population on a group level, besides income on an individual level^20–24^ such as in our previous study regarding living in an IDUD as a risk factor. The definitive mechanisms behind the association between income and mortality, both at the population level and in acutely ill patients, are poorly understood.^25^,^26^ Actual financial capacity can affect health, for example, in the ability to buy and access prescribed medication, as well as various kinds of follow-up visits, as in our previous and present results concerning annual income. However, it is more likely that it reflects a multifactorial background in which, in addition to financial capacity, health is affected by many other factors, for example, education, dietary habits and other lifestyle factors that we did not measure. In the previous study, we found that living in the immigrant-dense area of Malmö (IDUD) was an independent risk factor for mortality. However, this finding could not be replicated in the present study. This could be explained by the huge demographic changes in Malmö between the inclusion period of this study (2013–16) compared the previous study (2007). New urban areas have been built over the last 15 years, mixing populations with different SES within the previously defined IDUD/SDUD. In 2018, 44% of the population of Malmö comprised first and second-generation immigrants, compared to 37% in 2007. Many of the refugees from the Middle East and Afghanistan who have arrived in Malmö over the last decade are younger people. However, the patients in the ADYS study have a mean age of 69 (± 18.4) years. Thus, the ADYS study is not equally affected by the recent wave of immigration in Malmö. Country of birth as registered in this study was not associated with an increased overall mortality risk. We did not register country of birth in the previous study. Previous and ongoing smoking was a risk factor for premature death. In 2018 proportion of the general population in Sweden who smoke daily had decreased to 7% (Swedish National public health survey 2018 [Nationella folkhälsoenkäten, Hälsa på lika villkor], Public Health Agency of Sweden; personal communication, 2018. Even so, differences in smoking frequencies are still seen between different groups based on gender, education and income level, country of birth and employment. A higher proportion of people who stated daily smoking has been reported among people born in another European country (11%) and among people with the lowest income. A study from The Netherlands regarding Moroccan, Turkish and Surinamese immigrants also showed higher smoking frequencies among low SES male immigrants, and among high SES female immigrant.^27^ Nevertheless, we believe that information on income, as well as multiple comorbidities, smoking, high METTS priority and high level of dyspnea provide important clinical information that can guide ED physicians to recommend closer monitoring, and possibly a higher level of care in an acute setting, as well as closer follow-up in longer-term settings, in the same way as current diagnoses, level of dyspnea and triage priority. Importantly, although the effect size of each of the independent risk factors for mortality which we describe confers rather high relative risks, they should not be evaluated separately. Instead, we suggest that clinical decision-making may be primarily affected if several risk factors cluster together. However, the addition of information on annual income in this kind of clustering may improve the overall risk prediction.

## Limitations

We acknowledge several imitations. Patients were included only during the day on working days. Patients with high acuity or deranged consciousness probably went directly to the ICU and were therefore not included. The representativity of our study population, collected during daytime only, might be reduced as compared to if we had included patients during 24 hours. The lack of validation of using the modified NYHA class for grading dyspnea severity in acute ill dyspneic patients is a limitation. Moreover, in the ADYS cohort we registered if patients were born in Sweden, in the EU or outside the EU, rather than the actual country of birth. The actual country of birth might be a better risk indicator but consists of multiple small groups and is therefore difficult to handle statistically. Moreover, we have no information on the educational level of our patients, which may be a mediator between low income and mortality. We do not have information on employment status. Incomes from social security, various forms of pensions (etc.) in unemployed/retired individuals are included in the variable “income”. We agree that similar income from social security vs from an employment may differ in terms of socioeconomic burden.

Even though our study included around 800 patients, an even larger study cohort would be desirable to be able to detect exposures with smaller effect sizes.

## Conclusions

In concordance with a previous pilot study, in this study we have demonstrated that a low annual income is a strong and independent risk factor for long-term mortality in patients seeking care due to acute dyspnea. We have also demonstrated that the degree of dyspnea, triage priority, smoking habits and multiple comorbidities are independent risk factors for the same outcome. Our findings may improve the identification of patients at high risk of worse outcome already in the ED, thereby contributing to individualising the level of care, treatment and follow-up both in hospital and after discharge from the ED.

## Abbreviations

ADYS, Acute Dyspnea Study; AF, atrial fibrillation; CHD, coronary heart disease; CHF, cardiac heart failure; COPD, chronic obstructive pulmonary disease; CVD, cardiovascular diseases; ED, emergency department; EU, The European Union; ICU, intensive care unit; IDUD, immigrant-dense urban district; METTS, Medical Emergency Triage and Treatment System; NYHA, The New York Heart Association (NYHA) Functional Classification; SCB, Statistiska Centralbyrån, Statistics Sweden; SDUD, Swedishborn-dense urban districts; SEK, Swedish Kronor (the Swedish currency); SES, socioeconomic status; SoS, Socialstyrelsen, The Swedish National Board of Health and Welfare; SUS, Skånes Universitets Sjukhus, Skåne University Hospital.

## Data Sharing Statement

The datasets generated and/or analysed during the current study are available from the corresponding author on reasonable request.
